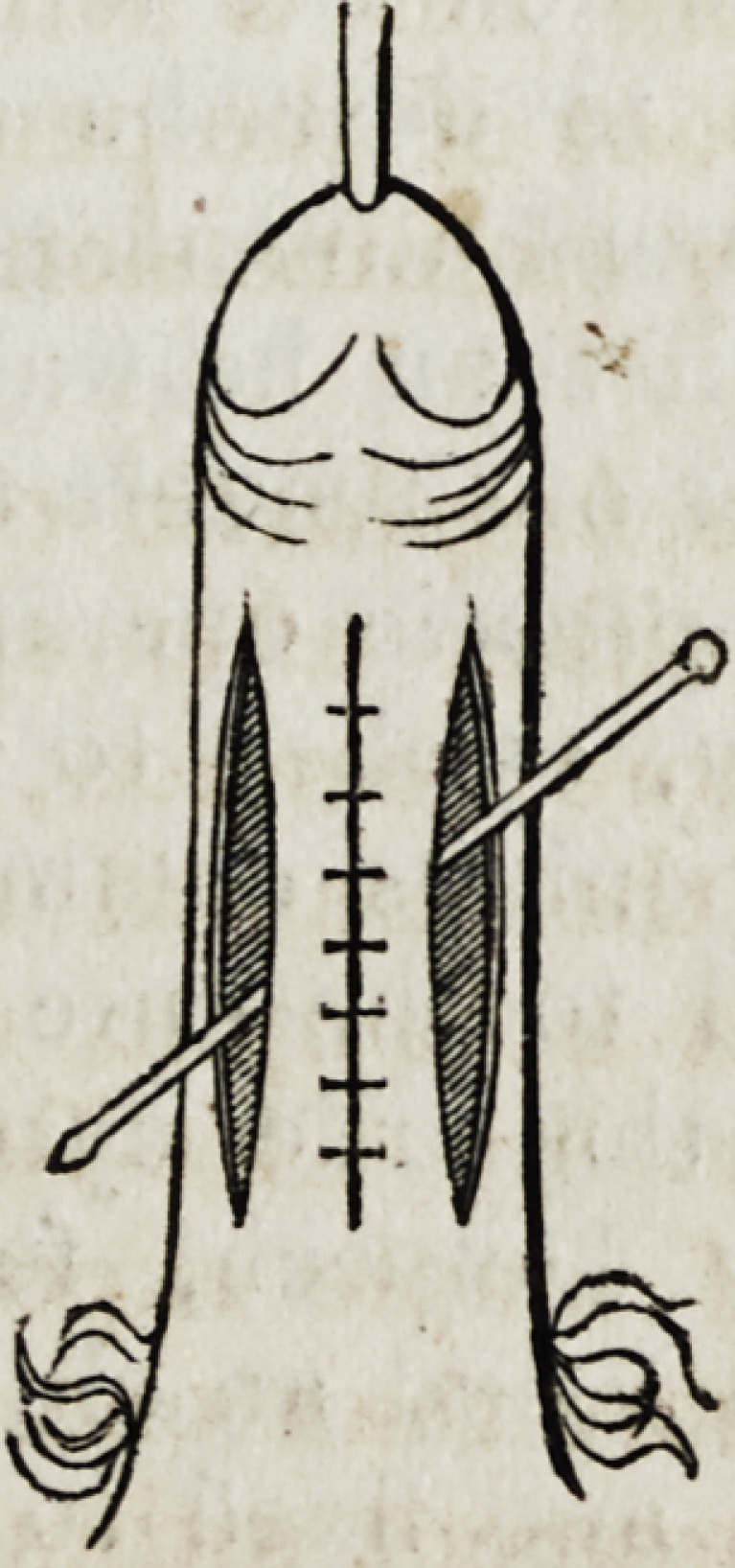# Autoplastic Surgery, or the Restoration of Parts of the Body Which Have Been Destroyed, by Borrowing from Other Parts More or Less Distant

**Published:** 1839-04

**Authors:** 


### ART. IV.

1. *Autoplastie, ou Restauration des parties du corps qui ont été détruites, à la faveur d'un emprunt fait à d'autres parties plus ou moins éloignées.* Par PH. FRÉD. BLANDIN, Chirurgien de l'Hôpital Beaujon, &c.—*Paris*, 1836.

*Autoplastic Surgery, or the Restoration of parts of the Body which have been destroyed, by borrowing from other parts more or less distant.* By P. F. BLANDIN.—*Paris*, 1836. 8vo. pp. 266.

2. *Handbuch der plastischen Chirurgie.* Von EDWARD ZEIS, Doctor der Medicin und Chirurgie, praktischem Arzte zu Dresden, &c.—*Berlin.* 1838.

*Manual of Plastic Surgery.* By Dr. EDWARD ZEIS, of Dresden.—*Berlin*, 1838. 8vo. pp. 576.

3. *Chirurgische Erfahrungen, besonders über die Wiederherstellung zerstörter Theile des menschlichen Körpers nach neuen Methoden.* Von J. F. DIEFFENBACH.—*Berlin*, 1829, 1830, 1834. IV. Abtheilungen. 8vo. und 4to.

*Chirurgical Essays, more particularly on the Restoration of lost Parts by new methods.* By J. F. DIEFFENBACH.—*Berlin*, 1829-1834. Four Parts. 8vo. and 4to.

4. *Practical Surgery; with one hundred and thirty Engravings on wood.* By ROBERT LISTON. *Second Edition.—London*, 1838. 8vo. pp. 529. (Chap VIII. *Restoration of lost Parts.*)

THE subject of the works before us is one which, although it can boast of an origin by no means recent, is yet comparatively new amongst us; and of this we need no more convincing proof than the fact, that the first two volumes on our list are the only works, in this age of book-making, which profess to treat of plastic surgery as a whole. It would be as difficult to find, as it is unprofitable to seek, a cause for the protracted slumber of this useful branch of the healing art, uninterrupted as it has been, save by the efforts of the Professor of Bologna and his immediate successors. Perhaps it is less difficult to assign a reason for its resuscitation with such renewed vigour in our own times. The rapid strides which surgery in general has lately made, and the degree of perfection to which the operative department in particular has been brought by the combined talent and boldness of modern surgeons, would naturally tend to the cultivation of every means of reparation, now that we appear to have learnt the extent to which we may carry the various forms of mutilation. It is not, then, so much with the object of entering into a critical examination of their contents that we have selected the works whose titles head this page, as to aim at presenting our readers with a concise view of the present state of plastic surgery in France, Germany, and England.

As the work of Dieffenbach touches only on parts of the general subject, and as Mr. Liston's treatise contains merely a single, short chapter, devoted to its consideration, we shall, in the following pages, only refer to them incidentally, taking as our guide the other authors, and especially Dr. Zeis, whose work, as might be anticipated, is considerably more copious than that of his French competitor. We shall examine the subject in both its general and special bearings; devoting to the latter division sufficient space to give a concise view of all the principal information supplied by our authors, and introducing a comparison between the practice of the three countries represented by them, whenever peculiarities or differences of opinion or practice seem to render this desirable. In general we shall abstain from criticism, and only interpose such remarks of our own as naturally suggest themselves under the practical point of view in which it is our especial object to regard the subject.

M. Blandin divides the main body of his treatise into six parts, as follows: 1. The sphere of application of plastic surgery. 2. The different forms of the same. 3. The modes of operation. 4. Requisite treatment after the operation. 5. Consequences of the operation. 6. The importance to be attached to the art. A general summary concludes the volume.

The work of Dr. Zeis is thrown into two grand divisions, in which the *general* and *special* parts of the subject are severally treated of. The first division is subdivided into ten sections: of these, one chapter is devoted to "the union of parts which have suffered complete separation from the body;" this is succeeded by a history of the Italian method of restoring lost structures, together with the modification introduced by Gräfe, termed by him "the German method;" then the Indian rhinoplastic operation is described; a separate chapter is next given to "the indications for plastic operations," which is followed by the consideration of the physiological and pathological conditions of transplanted parts, and the treatment, both surgical and medical, of patients: lastly, a section is devoted to general instructions respecting the operations; and this completes the first division of the work, which consists of about two hundred pages.

The second division is occupied with the various *special forms* of plastic surgery, and is comprised in nineteen chapters.

Dr. Zeis defines plastic surgery to be "that branch of operative surgery which has for its object the restoration of organized structures which have been destroyed;" and thus it is that, by art, the surgeon endeavours to imitate that which is the work of nature in the less complex of organized structures, and amongst the more simply organized beings. The amount of reproductive power appears, indeed, to bear an inverse proportion to the complexity of organization; or, to use the words of Zeis, "as the power of reproduction amongst the lower animals is more perfect than in the higher, so is the same law illustrated in different textures of the same individual; and the more complete and complicated an organ is, the more limited the reproductive power it possesses. Thus, in man, a hair or nail readily grows again so long as the root is unimpaired, but skin is replaced by a cicatrix which differs from the original structure; and severed muscle is united only by ligament. As we ascend to the more complex organs, we do not find that an eye, or a tooth, or a finger-joint are reproduced, as the claws or feet of a crab, when torn away, grow again."

Various terms have been applied to this branch of the healing art; a fact which Dr. Zeis does not fail to comment upon. We agree with him in opinion, that the nomenclature thus adopted by some authors is at once perplexing and useless, and even sometimes erroneous; as where the words "urethro-plastic" and "cysto-plastic" are employed to denote the simple operation for the healing of fistulous openings in the urethra and bladder. The addition of the adjunct *auto* (*αυτός, self*,) to the word plastic (a compound adopted by Blandin to signify that the new material was not derived from a foreign source, and intended as the antithesis of *Heteroplastic*), is vague and liable to misconstruction, as implying an innate power of reproduction. We are, therefore, willing to confine ourselves, with our German author, to the use of the simple expression "Plastic surgery,"* which is sufficient to imply generically all we mean: to such specific terms as do not convey an erroneous impression there can be no objection; of this class are *Rhinoplastic*, *Blepharoplastic*, &c.

It is well known to most of our readers that plastic surgery had India for its birth-place, where it was nursed by the care of certain low-caste priests, who derived their origin from the Brahmins, and upon whom the duties connected with the mysteries of astrology, instructing in military exercises, physic, &c. devolved; the subject of their operations being such unhappy criminals as had forfeited their noses to the outraged laws of their country. Whether the rhinoplastic operation spread thence over Asia, and whether the art of restoring lost parts of the body was known in Egypt, Greece, or Rome, seems difficult to decide. Galen, indeed, notices that the Egyptian priests knew how to make noses, but that they kept their mode of proceeding secret. Be that, however, as it may, Tagliacozzi or Taliacotius, (by which latinized name he is better known,) who was a native of Bologna, and was subsequently made professor of medicine and surgery at that University, became famous, in the latter part of the sixteenth century, for his success in the restoration of noses; a notoriety which was enhanced by the publication of his extensive and very learned dissertation, entitled *De chirurgia Curtorum per Insitionem.* This work was first published at Venice in 1597, and furnishes us with a curious specimen of the medical literature of that period; as it afforded its author an opportunity of exhibiting his widely diffused and varied information. We next find Cortesi, one of his pupils and professor of surgery in Messina, noticing the subject, and giving an account of a somewhat modified form of operation, in 1625:† and Hildanus relates how Griffon, a surgeon of Lausanne, restored the nose of a damsel cast by the chances of war into the hands of ruthless soldiers, who attempted to violate her: she, however, nobly resolved to sacrifice her beauty at the shrine of virtue, and actually cut off her own nose, (sure means, as our author observes, of cooling the lustful ardour of the assaulters.) In this instance the success was so complete, that the new nose was scarcely distinguishable as such, and the lady afterwards married. Molinetti, of Venice, likewise operated successfully in 1625. After this a long interval succeeded, in which plastic surgery made no progress: "silver and wooden

* Χειρουργία πλαςτικὴ (from πλασσω, to mould, to model in clay)—*Chirurgia plastica*, Plastic or Modelling surgery.

† Miscellaneorum medicinalium decades denæ. *Messinæ*, 1625. Fol.

noses became fashionable, as being deemed more convenient, and no one appeared disposed to follow the example of Taliacotius: thus exaggeration soon converted that which had lost its repute, into fable." (*Zeis*, p. 19.)

Taliacotius held it impractible to manufacture noses from the skins of other people, nor do we appear to possess any authentic accounts of such operation having been attempted. We, however, find the Italian professor setting forth the difficulties to be encountered in trying this vicarious form of operation, termed by Blandin "éteroplastie" for, as he observes, it would be no easy matter to keep two persons so nearly approximated and so perfectly at rest during a sufficiently lengthened period, as to effect the desired result of transplantation. The speculations on the practicability of this form of operation afforded considerable amusement during the interval that plastic surgery was at discount; and thus a ludicrous tale of J. Bapt. van Helmont gained much credence. He relates that Taliacotius operated for an individual by transplanting skin from the arm of a porter or slave on to the noseless face of his patient: that all went on well for the space of thirteen months, but at the end of that period the borrowed organ gradually lost its temperature, and in a few days became gangrenous: upon enquiry, it was found that at the self-same period the original owner of the nose had died. This fiction was no doubt the source whence Butler derived his well-known illustration in the first canto of Hudibras, although he chose to give the adscititious organ a local origin different from that assigned by Van Helmont.*

Plastic surgery, then, remained in this unsatisfactory condition until the latter end of the last century. At this time it would appear, according to the Madras Gazette of 1793, that the rhinoplastic operation was successfully practised upon some unfortunate individuals who had been mutilated after falling into the hands of the Sultan Tippoo. The operator came many miles for the purpose, and, as we are informed, possessed an hereditary title to the art of nose-making. A representation of one of his patients, before and after the operation, appeared in the Gentleman's Magazine in 1794. In 1803, our countryman, Lynn, attempted the operation, but without success; and in 1814, Mr. Carpue had his first successful operation: this was succeeded, in the following year, by another, which likewise terminated favorably; and the history of both these cases was published by Mr. Carpue in 1816. The cause of mutilation, in the former instance, was the abuse of mercury; in the latter, a sabre wound. Since that period, plastic surgery has flourished here and abroad; and, in spite of the claims of our German

* "Thus learned Taliacotius from
The brawny part of porter's b——
Cut supplemental noses, which
Would last as long as parent br——;
But when the date of Nock was out,
Off dropt the sympathetic snout."

The same facts and alleged facts afforded illustrations to many other satirical and witty poets. Thus Voltaire,

"Ainsi Talicotius,
Grand Esculape d'Etrurie,
Repara tous les nez perdus
Par une nouvelle industrie."

author for his native country, we do not think that our surgeons have been slothful in doing their part towards forwarding this useful and ornamental part of the healing art.*

The third section of Dr. Zeis's volume is occupied with matter rather foreign to the general object of the work, viz. "the reunion of parts which have been totally severed from the parent trunk." Under this head are enumerated the most remarkable cases of separated thumbs, fingers, noses, teeth, &c., being readapted after an interval of even many minutes' isolation. Of the truth of many of these no doubt can exist: indeed, we ourselves have witnessed one instance of this kind in the case of a finger. Next succeeds a description of the various modes of operating for the restoration of lost parts; and of these sections we shall give a short comparative abstract.

Taliacotius limited himself to the furnishing of supplementary noses, ears and lips; and he does not make mention of having attempted any other form of the plastic operation: he appears to have regarded the difficulty of restoring lips as by far the greatest. In renewing ears he did not employ the skin of the arm, but borrowed integument from the neighbouring part of the nape of the neck; thus copying, in this instance, the Indian method, which he never, however, adopted in the rhinoplastic operation. Taliacotius seems to have been fully impressed with the value of the art he practised, and did all in his power to supply its deficiencies: he notices accurately such imperfections as want of form, contraction of the nostrils, &c., and discusses the defects of colour, softness, and absence of sensibility. He further gives very judicious advice respecting the propriety of selecting fit subjects for operation; and strongly insists upon the necessity of deferring to operate in cases where the mutilation has arisen from a specific disease, until all evidence of its further extension shall have been eradicated.

The rhinoplastic operation which Taliacotius practised was somewhat complex, consisting of at least six distinct and independent divisions, viz. 1. The separation of the flap of skin from its *surface* of attachment (to the arm.) 2. The further division of the flap on its third side, so as to leave it only connected by a narrow peduncle to the arm. 3. The adaptation of the flap to its new position, the root of the former nose. 4. The total separation of the flap from the arm. 5. The formation of the nostrils and septum. 6. The attachment of the septum to the upper lip. An interval of some days, or even longer, was permitted to elapse between each of these steps of this tedious operation. The folio Venetian edition of the "Chirurgia Curtorum" is embellished with full-length sketches of individuals whose noses and lips are being reproduced by this "progressive developemental process," as our anatomists of the present day would call it. The left arm was preferred as being least inconvenient to the patient, and that portion of integument covering the inner margin of the biceps in the upper arm, was selected as best suited for the purpose. The requisite length and breadth

* We would here remark, that the greater bulk of Mr. Carpue's publication is occupied by "historical and physiological notices," matter which both Blandin and Zeis have evidently availed themselves of in their own historical compilations, without a due acknowledgment of the original source. An Account of two successful Operations for restoring a lost Nose. By J. C. Carpue. London, 1816. 4to.

having been, if desirable, previously marked out with ink, a longitudinal incision was made on either side of the intended flap, and the skin was separated from its connexions beneath by means of a bistoury; a bandage of linen being subsequently introduced beneath this bridge of integument to prevent reunion. The parts were then left in this condition, suppuration being promoted as tending to a speedy cicatrization of the under surface of the aforesaid flap; and, about the fourteenth day, when the surrounding margins had contracted and the partially isolated skin had assumed a more solid character, the second operation, which left the flap only connected by a narrow root, was performed. This step admitted of a modification according to the intention of the operator: where the aim was to restore the nose or upper lip, the peduncle was left below; but where the lower lip was the object of operation, the flap was left attached above, or nearer to the axilla.

One remark of the originator of this operation we cannot forbear from quoting, because we deem it well worthy of the observation of modern rhinoplastic operators. "The surgeon (he says) must be prepared for every emergency, and must especially take care that the adscititious parts are rather superfluous than deficient in quantity."* Attention to this point is likewise particularly enforced by Mr. Liston, (Practical Surgery, p. 229,) who appears to have been very successful in his rhinoplastic operations, as we may judge by the sketch of his first case, in which the new organ is represented as being "literally a very passable nose." Closer adherence to this rule would, we are persuaded, save much disappointment in the results of cases, where an insufficient allowance is made for the retraction which necessarily attends the cicatrizing process: and we are disposed to attribute much of the eminent success of Professor Dieffenbach in these cases to a just appreciation of this important point.

Another fortnight having been allowed to elapse, and various preparations, such as having the head and beard shaved, putting on a proper dress, getting the bowels freely moved, &c. having been effected, Taliacotius proceeded to the third step of his process, that of fixing the embryo nose in the position it was in future destined to occupy. This was achieved by means of sutures, and the arm, which had been previously supported by assistants, was now fixed by complicated bandages.† The subsequent separation of the remaining attachment of the flap from its place of growth, with the production and fashioning of the nostrils and septum, occupied the other three operations, which were accomplished at intervals, varying according to circumstances.

From Taliacotius's own account of his operation, we may justly conclude that he was a careful and skilful surgeon. Such likewise appears to have been the opinion of Gräfe, whom we find operating, in 1816, according to his directions.‡ The subject of Gräfe's experiment was a young man, twenty-eight years of age, who had lost all the cartilaginous portion of his nose by a sabre-wound, two years previously: the only

* "Præstat tamen semper in omnem eventum attentum esse, et ut redundet potius quid quam deficiat providisse."

† Of these and the instruments employed there are likewise various representations in the great work.

‡ Rhinoplastik, oder die Kunst den Verlust der Nase organisch zu ersetzen. Von C. F. von Gräfe. Berlin, 1818. 4to.

variation in this operation from that of the Italian professor was the combination of the last two divisions into one: the case terminated successfully. In the following year (1817) Gräfe adopted an improvement upon the Italian mode of operating, to which he gave the title of the "German method." This modification consisted in simply dividing the operation into two parts; the first comprising the separation of the required integument from the arm, except at one point, and its attachment to the face: by the second operation the process was completed. He further improved upon the confining bandages of Taliacotius, and also called in the aid of other mechanical means, such as compression, &c., in shaping the new organ and dilating the nostrils. His first essay of this sort was made on a girl who had lost her nose, when young, by malignant ulceration, (*Op. cit.* p. 174:) in six days the arm was liberated, and on the fourteenth the operation was completed. This material abridgment of the Italian operation was clearly of considerable importance as regarded the comfort of the patient, as well as the probability of ultimate success; but both have yielded to the more popular Indian operation, which we now proceed briefly to notice.

By the Indian method is understood that form of rhinoplastic operation in which the nose was derived from the forehead, in contra-distinction to the Italian fashion, by which the integument required was translated from a distance. It is true that Taliacotius constructed ears from the neighbouring skin of the head or neck, without possessing any knowledge of the Indian mode of proceeding; but he does not seem to have contemplated the practicability of forming a nose from the skin of the forehead. It would appear, according to the account of the operation performed at Poonah, which we have already alluded to, that the following was the course pursued: The line of incision having been marked out by the aid of a flattened wax model, the skin was divided in its circumference, so as to leave but a single isthmus of attachment between the eyebrows; the root of the nose and upper lip were next prepared by the requisite incisions, and the skin, being then raised from the forehead, was, by a half-twist, adapted to its new position; sutures were dispensed with, and the new relation maintained by dressings. The patient was thus kept in the recumbent posture for about five or six days; and on the tenth a plug of soft lint was passed in, to support the nose, and keep the external aperture free. It does not appear, however, that there was any septum: one single outlet, therefore, existed, in place of nostrils. (See Carpue's cases, p. 16.) We are further informed that, in India, they were likewise acquainted with the operation for restoring both ears and lips. The improvements which Mr. Carpue made upon the above method were the following: He *commenced* his operations by making the necessary excisions for the reception of the new flap; he added the septum, and employed sutures in retaining the opposed parts in contact; lastly, he endeavoured, as much as possible, to approximate the margins of the wound of the forehead. Gräfe, in Berlin, was the next to patronize the Indian fashion, and his first case occurred in 1817: the subject was a female, fifty-one years of age, on whom the operation proved successful. His example was followed by Walther, Rust, Chelius, Fricke, Dieffenbach, &c.

In France (singular fact in that land of personal vanity) plastic surgery made slower progress. Thus we find, in 1819, that the rhinoplastic operation was decried, as offering very little compensation for the suffering endured in procuring a new nose of flesh, where one of metal or wood answered the purpose so well.* It appears that M. Thomain, of Aix, was the first to perform the Indian operation in France, which was soon imitated by Dupuytren, Lisfranc, Delpech, Velpeau, Labat, Blandin, &c. In regard to England, it is curious to remark how limited a part Dr. Zeis makes our surgeons play in forwarding the interests of this his favorite art. The only names he mentions as deserving of notice, until very recently, are those of Carpue, Hutchinson, and Davies: whereas, we would venture to assert that there is scarcely a London or provincial hospital surgeon who has not performed various plastic operations of more or less importance. Either these cases (and many have come within the compass of our own observation) have not been deemed of sufficient importance to merit publication, or our author has failed to search, with wonted German assiduity, the English medical journals:—however, our business lies at present with continental surgery; the vindication of our national claims must, therefore, be left to other hands.

That "necessity is the mother of invention" appears to be a reasonable mode of accounting for the fact that India was the birth-place of plastic surgery; for the mutilating punishments to which adulterers, deserters, and various malefactors rendered themselves obnoxious yielded frequent opportunity for the exercise of the art in question. In Europe, disease has done what the cruelty of the law did in India; and syphilis, lupus, scrofula, &c. have made cases whereon to exercise the ingenuity of the plastic operator. The principal part of the seventh section of Dr. Zeis's book is occupied by remarks, connected with this subject, upon the most fitting cases and periods for operations, with the relative advantages derived from various age, temperament, &c. These observations are, for the most part, judicious, although many are so self-evident as scarcely to be needed in a work like the present. "The greatest variety," he observes, "of form which maimed noses present results from injuries, such as cutting, biting, burning, &c.;" but the cases which he deems the least favorable for operation are those in which the loss of structure has been from carcinoma: "indeed," he adds, "we are scarcely warranted in subjecting patients, especially old people, who are labouring under this disease, to a painful operation, which has so little prospect of success." (p. 121.) In this section, likewise, Dr. Zeis enters into a review of the various indications for plastic operations in different parts, preliminary to entering upon the subject in detail. As regards age, he remarks, "the necessity for plastic operations may occur at all periods of life, but less frequently in children than at a maturer age. The tenderness and susceptibility of childhood for the most part contra-indicate the operations in question; and, further, the consideration that a well-proportioned and suitable nose for a child would be misplaced on the face of an adult, would render it preferable to defer the rhinoplastic operation until, in maturer years, the face and head have become more perfectly developed." (p. 137.) In the very aged these operations are not excus-

* See Dict. des Sciences Médicales; art. *Nez.*

able, unless for the purpose of obviating annoyance or suffering. Erysipelas is a justly dreaded source of interference with the success of plastic surgery; therefore, there ought to be some circumspection used in selecting for operation individuals who have been subject to this affection, and extra precaution employed in the after treatment. But we shall return to this subject anon.

In his observations on the applicability of different portions of the skin to the purposes of transplantation, Taliacotius divides the common integument into four sorts: 1, that which is devoid of hair, and of limited pliability, as in the palmar and plantar regions; 2, that which is acted on by a superficial muscle, as the scalp of the forehead; 3, that which has muscle closely and intimately connected to it, as in the face; 4, the general integument of the trunk and limbs. It was skin of this last kind only that Taliacotius regarded as suitable for transplantation; whereas, on the contrary, surgeons of the present day consider the forehead to afford the best. Blandin so far differs in opinion from the Italian operator as to prefer that integument which is more fixed, and beneath which the cellular membrane is scanty and deficient in laxity: he calculates that such skin produces the firmest and most consistent flaps, and is even better pleased to have an aponeurosis which he may raise with the skin. (p. 97.) In this opinion Dr. Zeis does not altogether concur, but allows that integument for transplantation cannot be too thick. It is true that dense skin is less liable to shrink, and retains its form better; but there are doubtless some cases requiring operation, in which a thinner skin would be the more available. The most important point, however, gained by substance in these cases is the better prospect of union, owing to the relatively greater number of the supplying vessels. Of this fact the usual success of the Indian form of rhinoplastic operation is an evidence; and it is further remarked by Dr. Zeis, that he always augured a favorable termination when the scalp of the forehead was thickly studded with sebaceous follicles, which he considered as indicative of corresponding density and richness in vessels. (p. 153.) He likewise considers the facial integument as eligible, if requisite; but justly remarks that the proximity of the eyes, mouth, &c. forbids any extensive removal of skin from this region. If the scalp behind the ears be employed, it is recommended to shave the part first, and subsequently, as the hairs present themselves, to withdraw them with a pair of tweezers.

The following remarks are interspersed through the succeeding pages of Dr. Zeis's work. *Contraction* is inevitable to a certain extent, and in some instances to a very considerable amount, in transplanted flaps: the increase of thickness is, in these instances, nearly proportioned to the decrease of surface; an effect which, though often advantageous, by no means compensates for the accompanying evil: moreover, it is important to make allowance for the probable shrinking, in order to aid, as much as possible, the adhesive process, by avoiding the mechanical impediment of dragging, which would alone be sufficient to offer a material obstruction to this all-important result. *Loss of temperature*, more or less, is also a necessary consequence of "peninsulating" a portion of integument; and this effect is proportioned in intensity to the natural amount of organization of the flap, and the extent of original connexion left to it in its new relations. This condition does not, however, commonly endure beyond the first hour or two; and it is not unusual to find the temperature raised on the following day, even above the natural standard. The *pallor* which accompanies the reduction of temperature is attributable to the absence of blood from the vessels. This state is often converted, in the course of a few hours, to one of an opposite nature: the vessels become gorged, and a livid hue supervenes, which is dependent on the sluggishness of the venous circulation. Where this turgidity is very great, mortification is to be dreaded; and Dieffenbach has, under such circumstances, drawn blood from the flap by the lancet or by the aid of leeches.* The recommendation of Blandin to include, if possible, a considerable artery in the peduncle of transplanted skin is, therefore, rather short-sighted; inasmuch as nothing would be more likely to favour the state above described than a large supply of arterial blood, without ensuring its speedy return. The French author, however, inveighs strongly against the practice recommended by Dieffenbach, and declares the result of his own experience and observation to be directly opposed to that of the Berlin professor: he further cites, as coinciding authorities, Delpech and Serre; the latter of whom ascribes the more frequent occurrence of gangrene in the Italian operation to the want of a direct and large supply of arterial blood. (*Blandin*, pp. 97, 105-7.) These are points of by no means trifling interest to the plastic operator. Our own observation disposes us to adopt the opinion that mortification more frequently results from an over-charged state of vessels than from a want of power to sustain vitality. It is, of course, of great importance to distinguish between the lividity resulting from repletion, and that which is the consequence of a deficient influx of blood; a point which has been justly commented on by Mr. Liston, (*Practical Surgery*, p. 229 :) in the latter condition, he observes, "the surface is cold, the lividity being antecedent to gangrene, and removal of blood would but hasten destruction of the part." As union by the first intention is the most desirable object, it becomes essential that the operator should direct towards it his chief attention. For this purpose Dr. Zeis seems to lay great stress upon the importance of moderating inflammation in the neighbourhood of the line of incision: he employs cold applications, which, he observes, are more grateful to the patient; and recommends, if need be, the adoption of the most active antiphlogistic measures, to obviate the suppurative process. (pp. 163-4.) "If, however," he adds a little further on, "adhesion have taken place only at some points, it does not signify that the cure be subsequently completed by granulation and suppuration." A very annoying event, short of total failure, is partial gangrene: when this occurs in the septum, it is of the least importance, as a fresh one may be procured from the upper lip. If there be no appearance of union by the first intention before the third or fourth day, but in its place general suppuration, then, says Dr. Zeis, we may conclude that the operation has been in vain, and that gangrene is inevitable. After the sixth day, Blandin ascribes gangrene to inflammation: it is then, he remarks, of a moist character. (*Blandin*, pp. 210-11.)

The phenomena connected with the *sensibility* of a raised flap of skin are such as might be anticipated. The subject of the operation is per-

* Chir. Erfahr. Abth. ii. p. 72.

fectly aware of the contact even of the finger, (always supposing the case is proceeding favorably;) but, naturally enough, refers the sensation to the original seat of the transplanted integument: thus, in the common rhinoplastic operation, the contact of any body with the new nose is felt in the forehead. Habit, however, corrects this erroneous impression, (if such it may be termed,) and in the course of a few weeks the new organ ceases to require a vicarious performance of this office, and establishes for itself the right of internal recognition, and of communicating directly with the external world. The sensibility is usually more or less perfect in proportion to the extent of the connecting isthmus; yet it has been observed by Gräfe, that in one case of rhinoplastic operation from the forehead, which he witnessed, although there was total annihilation of sensibility, the processes of granulation and cicatrization proceeded, nevertheless, with extreme activity.* At the end of a long case described by him, Blandin remarks that no sensation was experienced in the new nose until the healing process was quite completed; and that then it returned, together with the sense of smell, which had before been quite lost. (*Blandin*, p. 134.) In speaking of *erysipelas*, Dr. Zeis remarks, "this disease is of frequent occurrence after plastic operations, especially of the face. The general nature of the operation, the presence of sutures, &c., aid in producing, the day following the operation, an inflammatory condition, which sometimes merely resembles, but more often really is, erysipelas. Where any predisposition to this disease exists, general precautionary measures must be attended to; as, in correcting secretion, if irregular, keeping the bowels free," &c. The propriety of avoiding the operation at a period when this epidemic is prevalent, as is sometimes the case in the wards of our hospitals, is too self-evident to be insisted on.

It so happens occasionally that it is requisite to transpose the relation of skin and mucous membrane; as in turning up a septum nasi from the upper lip: it is interesting to observe, in such cases, how readily the latter adapts itself to its new and exposed position: it rapidly assumes, as is known to every surgeon, the character and function of common integument; indeed, the converse will occur, although less perfectly. The anatomical examination of parts which have been subjected to operation has not presented anything worthy of remark: the skin had lost none of its healthy characteristics, and the cicatrix was like other cicatrices. In a case examined by Gräfe, the supplying vessels from all sides were, as might be anticipated, much enlarged.

The *surgical* and *medical treatment* of his patients forms the ninth section of Dr. Zeis's work. It contains no peculiar views: we shall, therefore, pass it by with one or two quotations. In regard to *diet*, Taliacotius required his patients to live abstemiously on the first day, but generously during the rest of the week: he, however, recommends that the process of mastication should be avoided, as interfering mechanically with the adhesive process. Occasionally, spongy and coarse granulations will spring up, threatening to burst the sutures: in such case, the same author recommends the use of astringent washes and ointments. Pain in the scapular region was, with him, a frequent source

* Gräfe und Walther's Journal, Band xii. p. 11.

of troublesome complaint: this, however, arose solely from the awkward and constrained position of the arm. When the inflammation has exceeded the healthy (or, more properly speaking, desirable) bounds, Gräfe says the most vigorous antiphlogistic measures, general and topical, must be employed, if requisite. He notices erythematous inflammation as very troublesome and painful. Stimulating applications, such as aromatic and spirituous fomentations, have been occasionally used to aid the circulation; but Dr. Zeis has never seen any case in which their employment was needed. With respect to the ordinary dressings to be applied after the completion of an operation, we fully coincide with Mr. Liston in the opinion that they cannot be too simple. A piece of lint, dipped in warm water, and a strip of oil-skin to preserve its moisture, is all that is needed, and very preferable to salves and plaster; the displacement and renewal of which may seriously interfere with, or even destroy, the tender adhesion of newly-adapted surfaces.* The medical treatment in these cases is, as a matter of course, analogous to that of patients who have submitted to other surgical operations.

The tenth section (a long one) treats of the *general forms of operation*. From this also we shall cull short passages here and there, previous to introducing our readers to the special operative division of the work. Dr. Zeis remarks, that "reparation may be effected by completely or partially separated flaps of skin;" and, again, a second division is said to be that "in which transplanted integument, without perfect separation, is derived from a foreign source or from the same individual." The doctor admits that the former of these operations has only existed in the imagination; and that, in spite of the authority of Van Helmont and Butler, flesh will most readily take fresh root where it is indigenous. In the latter operation, the required skin "may be derived from a contiguous or distant site;" and it may be desirable that such flap, when removed to its new position, should "adhere either by its whole surface or by its margin only:" for the cure of fistula the former is needed; the rhinoplastic operation is an instance of the latter. The after-section of the peduncle is a modification due to Dieffenbach, but deprecated by the French authorities, Blandin and Labat. (*Autoplastie*, p. 124; and again, p. 172.) This difference of opinion gives occasion to Dr. Zeis to make some rather amusing strictures upon the former writer; in which he censures him for claiming, in the names of Lisfranc and Lallemand, the merit of improvements which justly (as we likewise believe) belongs to Dieffenbach. Indeed, it is not difficult to discover that our German author holds in no small contempt the opinions of his French contemporaries, at any rate on the subject of plastic surgery. The plan which Dieffenbach has adopted consists in causing the section on one side of his flap to be continuous with the excision for the reception of it. Thus, though in the original operation it signified not whether the flap was perpendicular or oblique to the long axis of the face, it is of importance in the above improved operation. The latter is preferable, as by its means the dragging in making the turn is slighter, reducing the 180 degrees of a circle

* We were never more fully impressed with the advantages of extreme simplicity of surgical dressings, than when attending the practice of Laugenbeck, the talented professor of Gottingen.—Rev.

to 140. A simpler operation, in which no twisting of the integument occurs, may be performed for the closing of fistulous openings, &c.; but, for this, skin must be sufficiently loose in its connexions; an incision must be made on three sides of a flap, which, being raised, is drawn forward, and adapted over the aperture to be closed.

Cases occasionally occur in which, as a consequence of destruction of the nasal bones and cartilages, the tegumentary covering of the nose sinks for want of support, without itself having suffered any injury. This condition Dieffenbach has proposed to remedy in the following manner: The nose must be divided into several—say three—flaps, of which, in such case, the central would be that covering the dorsum of the nose; slices are then removed from each interval, but of such a form that there shall be scarcely any loss superficially: thus, the principle is analogous to the formation of an arch by a series of wedges. An operation of an opposite character may likewise be required by loss of structure; viz. the insertion of a new portion from a neighbouring part which can better spare it. If the surface have been destroyed by disease, without injury to the soft parts beneath, then Dieffenbach recommends that a new coating should be borrowed from the forehead; and, again, the reverse condition (the deficiency of bone and cartilage) may also be benefited by the transplantation of a portion of skin from the same source: the object of this latter operation requires, however, a little explanation. The transplanted portion of scalp is laid between the separated nasal integument, and there allowed to fix itself, although it is ultimately destined to form the new bridge to the dilapidated organ. This object is effected by means of dissecting out, at several times, the surface of the transplanted skin, and by approximating the edges of the original tegument: thus is the deeper or cellular portion of the new structure allowed to recede, and form a vicarious support to the depressed nose. (*Erfahrungen*, Bd. ii. iii.) A similar operation is recommended by Mr. Liston. This surgeon employs one of two methods, according to circumstances; "either shaving off the surface of the depressed portion, or by establishing a sulcus in its centre, raising the integument a little on each side, and then inserting and permanently retaining a central portion brought from the forehead, sufficient to raise the part to its proper level." (*Practical Surgery*, p. 233.) This operation is thus completed at once. In the former case, where the nose has simply sunk from loss of its natural support, the same surgeon deprecates Prof. Dieffenbach's operation, and asserts that the proper treatment of these cases consists in "dividing any abnormal adhesions, and carefully stuffing the nose so as to retain the integument in a proper shape, until its disposition to fall is in part overcome, and firmness and stability are obtained. A septum may subsequently be formed, and the stuffing renewed until the cure is complete. (*Ib.* p. 234.)

In connexion with the above forms of disease, we may notice urethral and vesico-vaginal fistulous openings, for which Dieffenbach has successfully practised the application of the following form of suture: A curved needle, being armed with a strong ligature, is passed, stitchwise, around the opening to be closed: i. e. the skin or mucous membrane, as it may be, is exposed and treated as we should treat a common form of bag, to which we desired to apply a running string. The suture is passed in and out, its extremity terminating where it commenced; the ends are then knotted together, and the surrounding loose integument is puckered up, so as to close the fistula by tightening the suture; and this can of course be regulated or repeated at pleasure, as long as the thread holds together. The plaiting of the skin becomes permanent, and thus the cure is effected.

The accompanying woodcut, representing the application of this suture for the cure of urethral fistula, will convey the best idea of its mode of employment. The internal circle represents the pared margin of the fistulous aperture; whilst the external dotted circle indicates the stitching course of the suture: the extremities of the thread must be left sufficiently long and free to admit of their being tied with facility.*

It behoves us now to say a few words respecting *sutures* generally. The primary adhesion of newly adapted parts is, of course, mainly dependent upon the accuracy and sufficiency of the means employed for keeping them in contact. This result may be accomplished, as we have already had occasion to notice, without sutures: such was the Indian practice. Taliacotius employed the simple knotted suture. Mr. Carpue, in his operations, used the same form; applying five sutures, one being for the septum. Gräfe recommended the employment of small bars of ivory or boxwood, which were to be laid, with a slightly convex surface, upon the edges of the wounds, and presenting a plane surface for the sutures to be applied over. But the winding or twisted suture of Dieffenbach appears, according to Dr. Zeis, to have the preference given to it above every other in Germany. It is similar to that which we employ likewise in England; the method of application consisting in passing a series of needles through the skin, (its whole thickness, says Dieffenbach,) about one or two lines distant from the incised margins on either side: thick, unwaxed, woollen ligatures are then wound, in a circular or figure-of-eight form, round these, and subsequently secured firmly by a knot: the extremities of the needles are lastly cut off. Our French and German authors are here again at issue as regards the number of sutures; Zeis thinking, with Dieffenbach, that there cannot be too many; whereas, Blandin (p. 110) considers five sufficient for the nose, and that, in many plastic operations, they may be dispensed with altogether. Mr. Liston recommends that the needles be cautiously with-

* Reference may be made to this cut, in reading the description of the operation it is intended to illustrate, a few pages further on.

drawn on the third day, but "without disturbing the threads or the crust which has been formed about them." (*Ib.* p. 227.)

The second or *special* part of Dr. Zeis's work treats of the particular operations in plastic surgery. The first section of this division is occupied by the *Rhinoplastic* operation* in all its forms, and with all its varieties and modifications in detail. The amount of injury or loss which the nose may sustain from disease depends mainly upon the nature of the malady and the structures attacked. Further, the loss of structure may be the consequence of accident; in which case, likewise, the extent of injury is proportioned to the violence of the blow, or character of the implement by which the hurt is inflicted. We would therefore suggest, as a simple arrangement, to classify the conditions resulting from injury to this organ (for it is with results we have to deal) under three heads: 1. That in which the common integument alone is destroyed; 2, that in which the cartilages have also suffered; 3, that in which the bones are likewise implicated. There may, doubtless, be modifications of each of these conditions; i. e. they may exist independently of one another, or in combination. Where the latter is the case, the resulting condition necessarily is a total annihilation of the organ. We will not, however, anticipate our author, but allow him to speak for himself as we follow him through the somewhat diffuse pages of his work. Pass we on, then, at once to that form of defect, for remedying which surgical aid is most needed, viz. the deficiency of the whole or greater part of the nose.

Dr. Zeis remarks that "the greatest proportion of nasal imperfections which require operation do not belong to this worst form, of which we are now about to treat, but are principally of that class in which the anterior and cartilaginous portions of the nose are wanting; and where parts of the alæ nasi and septum remain, and are in a condition to be turned to useful account in the operation." (p. 266.) We need scarcely add that, where this is the case, the operation requires such modifications as will spare the scalp, as well as produce a better specimen of the operator's handicraft. The method adopted by Dieffenbach is approved of by Dr. Zeis: we shall therefore describe the operation which he recommends as the most desirable where the scalp is thin. He raises from the forehead an oval flap of skin, which has its connecting peduncle on one side of the median line, and adapts it, in the usual way, on or over the remnant of the former nose, confining it by means of the suture already described. The extremity of the flap is next cut up in two places, so as to form the septum in the centre and the alæ laterally; or this may be effected before the application of the sutures. The septum and upper lip are then connected, and the long sloping points of the alæ are turned in, and fixed by pins. "This method," says Dr. Zeis, "is found to present peculiar advantages where the forehead-scalp is thin; as the point of the nose thus gains greatly in strength and capability of resisting contraction, at the same time that the nostrils are prevented from growing together." (p. 270.) Labat has described a similar operation, but with the flaps cut out broader and rounder in front. Where the scalp is dense, Dieffenbach does not consider this operation applicable, because

* From Ῥὶν, ῥινὸς, the nose, and πλάσσω, to model; or πλαστικὴ, the art of modelling or making images in clay.

the alæ become too thick: indeed, it is in such case impracticable to turn the flaps in. That the operation succeeds admirably in the hands of Prof. Dieffenbach, when the scalp is not too dense, will be testified by all who have had an opportunity of witnessing his practice. This operation has also been adopted in England; but Mr. Liston prefers procuring the septum, in ordinary cases, from the upper lip; and for this purpose he divides the operation into two parts, the formation of the columna being effected after the body of the nose is united in its new position. (*Practical Surgery*, p. 225.) Having premised that it is useful as a guide to make a paper model of the intended nose, and subsequently to trace its outline on the forehead, Dr. Zeis proceeds to describe the different steps of the operation. This is, however, ushered in by a list of the requisite apparatus; but we will not detain our readers with a detail of the many minutiæ recounted, from the seating of the patient on the operating stool till his return to bed. Suffice it to say that the operation, as described by Dr. Zeis, follows the usual course. After the raising of the flap, and the requisite excision around the site of the former nose are accomplished, our author remarks "that the greatest possible care should be taken to arrest the bleeding; for there need be no hurry about fixing the flap, and it is even of considerable advantage to defer it for some time." (p. 276.) We do not quote these opinions as novel; we are aware, on the contrary, that the recommendation and practice are common, but we much doubt the expediency of the same. Our own observation has induced us to believe that too much importance is attached to the necessity of arresting all appearance of hemorrhage before adapting the fresh cut surfaces; and we feel satisfied that the vitality of the almost isolated flap is temporarily lowered by cold applications, which are commonly had recourse to; and the consequence of a reaction therefrom, tending to secondary oozing of blood or the justly dreaded congestion, is rendered more probable. If we be not mistaken, this opinion is acted upon by many operators, and Dieffenbach amongst the rest; at least, such is our recollection of this able surgeon's practice. In case of hemorrhage from small arteries on the forehead, torsion is recommended as the most ready means of checking it. As to the order in which the sutures are to be applied, Dr. Zeis prefers commencing by attaching the septum to the upper lip; and he is very earnest in urging the propriety of not being too sparing in the application of sutures; for, he adds, "I think it by no means advisable, as many operators do, to be contented with three sutures on either side of the nose;" and, further on, "should there be at any part space enough left between any two sutures, add even a small needle or a simple suture; for the parts must be accurately adapted, for union by the first intention to result." In this advice we fully coincide. Before dressing the forehead, Prof. Dieffenbach is, we believe, in the habit of applying two or three sutures, to assist a little in approximating the edges. It occasionally occurs that certain soft parts of the nose may be still in existence, although from altered position, resulting from the loss of support, they have ceased to be either serviceable or ornamental. Of such parts Dr. Zeis recommends, very properly, that the surgeon should make use, as an aid in renewing the whole organ; and relates a case of his own, in point, (p. 290,) which was successful.

The advantage of affording mechanical support to the newly-formed nose is discussed; and a case, which was treated by Mr. Tyrrell, of St. Thomas's Hospital, is narrated at length. The support employed in this instance was a temporary bridge of platina, which was subsequently removed, and proved of permanent benefit to the form of the organ. The employment of any permanent extraneous support is justly deprecated by modern authorities. (*Liston*, p. 227.) A series of cases then succeed, which we have not room to insert or treat of at length: but their very titles—while they infallibly suggest the idea of a cobbler's bill—attest the splendid triumphs of modern surgery:—"the reparation of a mutilated nose by the formation of a new bridge," (p. 306;) "the removal of a portion of the nose, for repairing this organ after being crushed," (p. 307;) "the restoration of the alæ nasi and lateral wall of the nose," (pp. 312-14;) "repairing minor imperfections in the alæ nasi," (p. 318;) "raising a depressed nose," (p. 321:) &c. &c.

In connexion with that division of the subject relating to "reparation and restoration of the alæ nasi," we may here notice an operation proposed and performed by Dr. Mütter, of Philadelphia. In his case the right ala had been destroyed by disease, and the septum on the same side was of course exposed. The form of operation resorted to was that described by Blandin, under the head of "Operation par glissement du lambeau." The first incision extended "from a few lines above the superior border of the orifice to a short distance below its inferior, and was directed downwards and outwards; the second commenced at the terminal extremity of the first, and extended horizontally outwards about an inch:" a triangular flap, three lines in thickness, was thus marked out and dissected up. The third incision extended "from the initial extremity of the first to the point of the nose;" a triangular piece of the cicatrix was then removed, and, the flap being brought forward, was fixed by the interrupted suture: a roll of oiled lint was introduced into the new nostril. The inclination of the septum to one side, resulting from after-contraction of the flap, was diminished by "dividing the line of union between the base of the flap and the cheek; and a subsequent semicircular incision in the same position gave the rounded margin of an original ala, care having been taken to prevent reunion of the cut surfaces. This operation possesses the advantage of not requiring torsion of the flap.*

Under the head of "Means for amending depressed noses," Dr. Zeis describes an operation (also originating with Dieffenbach) for rectifying a condition which accompanies the double harelip with fissured palate. In these distressing cases the nostrils present an expanded appearance, and the septum is displaced in such a manner as, after the operation for the harelip, to prevent the nose from rising to its proper position. "The division of this fold removes the deformity. For this purpose the head is fixed, and the fleshy septum seized and drawn to one side, until the folded margin of the cartilaginous septum is brought into view; it is then pierced by a small scalpel, and the whole extent of the internal septum slit up, even to its junction with the osseous partition. The point of the nose then rises unaided, but may be still further elevated, if

* American Journal of the Medical Sciences. 1838.

requisite, by mechanical means." (p. 326.) This position it is necessary to preserve, in order to prevent reunion in the previous form. Granulations will complete the cure.

The restoration of the septum may be accomplished from the nose itself or from the upper lip: we shall briefly notice these two forms of operation. The conditions which Dr. Zeis recognizes as favouring the former are the following: "a nose so large that the removal of a portion of it would tend rather to improve than detract from its beauty; or, further, an upper lip which is low or scarred." For the purpose of executing this operation, "a slice from the whole thickness of the nose is raised, about an inch in length and four or five lines in breadth, pointed above, and having its connecting pedicle below at the tip of the nose: this is turned and made fast, in its proper position, to the upper lip." The margins of the interval left are then brought together by suture. (p. 331.) The accompanying woodcut represents the sections, and the manner of the subsequent adaptation: the upper extremity of the flap, as low as the transverse line, is sacrificed as useless.

A modification of this has been proposed by Dieffenbach as peculiarly applicable to "turn-up, saddle-backed" noses; viz., instead of twisting the pedicle, the lateral incisions are carried on "to the free margin of the wide-spread nostrils. The inferior extremity of the flap being then pared in preparation, the whole is liberated, at its sides and upper parts, from the subcutaneous cellular tissue; the most depending portion alone being left, with its original connexions, for the purpose of preserving its vitality. This is then usually found sufficiently moveable to allow of its being drawn down into the proper connexion with the upper lip; but, if requisite, the cartilaginous portion of the point of the nose and septum must be cut across to a sufficient extent to allow of the required adaptation." (p. 332.) The rest of this operation is similar to the last described. The case, however, may be reversed; viz. the nose may not be able to spare a flap, and the upper lip may be even improved (as in strumous subjects) by the abstraction of a portion from its centre: such flap is then connected to the posterior surface of the inferior margin of the nose. We have seen the appearance remarkably improved in this twofold manner by an operation of this nature. If preferred, the septum may be simply turned up, as the mucous membrane soon takes on a cuticular character; or the mucous membrane might be left undisturbed if the upper lip were scanty. A further method of performing this operation is by removal of an oblique portion of integument from the upper lip: this, however, leaves an uglier cicatrix. Apertures in the nose may be closed by simply paring the edges and adapting them, or by a transplanted flap. Dr. Zeis, in conclusion, gives various hints for the after-improvement of newly-formed noses. These it is not essential for us to notice; but we would venture to recommend attention to one point, which might render such emendations occasionally less essential: we allude to the propriety of making the receiving surface for fresh-cut margins sufficiently broad. The scar is thus rendered less perceptible, because the depression is less marked; and, moreover, the probability of ready adhesion is, in a certain degree, proportioned to the extent of surface brought in contact: but the golden rule is, as Dr. Zeis observes, "to keep constantly in view that the longitudinal diameter of the wound should correspond accurately with that of the nose." (p. 345.) In many cases, Dieffenbach has performed the after-operation of removing the cicatrix from the forehead, and re-approximating the edges with sutures. Even a section on each side has been found to succeed, in like manner, when the above dissection has been objected to.

By the *Blepharoplastic* operation* is understood that branch of plastic surgery which has for its object the restoration of lost eyelids. In the older works which treat of plastic surgery, no mention will be found of this particular operation; and it is only since the resuscitation of the art during the last century that it appears to have been practised. Under this head are usually included sundry minor operations for the cure of ectropion, lagophthalmos, &c. The importance of the relief obtained by the successful treatment of these truly serious affections will be sufficient apology for our dwelling a short space on this division of our author's subject.

The blepharoplastic operation presents features of peculiar difficulty to the surgeon, on account of the very complex organization and delicate texture of the structure to be replaced ; but, as Dr. Zeis observes, "it is impossible to produce an artificial eyelid from the common integument, which shall possess mucous membrane, muscle, cartilage, glands, ciliæ, cellular tissue, and so forth;" therefore, we must be satisfied to imitate nature as nearly as possible in the form of the protection which it is our object to supply to the tender organ that has been deprived of its natural covering. The first plan which Dr. Zeis notices is that proposed by Dzondi, in his "Contributions towards the Improvement of Medicine," for rectifying that shortened state of the eyelid which results from a contracted cicatrix. It consisted simply in dividing the cicatrix, and allowing it to heal by granulation, so that the broader scar might remedy the defect. The objection to this mode of proceeding is, that, whatever care may be taken during the period of healing, the after-contraction will, as Dr. Zeis observes, in almost every case, negative the anticipated benefit. The same surgeon (Dzondi) afterwards suggested an improved operation, which he details in Hufeland's Journal for 1818, p. 99. We shall proceed to notice the methods adopted by Dr. Fricke, of Hamburg, and Prof. Dieffenbach. Dr. Fricke's operation is peculiarly adapted for extreme cases of ectropium; and where the conjunc-

* From Βλέφαρον, the eyelid, and πλαστική.

tiva has, from exposure, become thick and cartilaginous, he recommends its removal some days previous to the restoration of the lid.* A case will best illustrate his mode of treatment.

"The subject of the operation was a man sixty-three years of age, in whom the upper eyelid was held firmly back and everted, after the healing of a severe burn: the amount of the eversion caused the points of the ciliæ to be intermingled with the eyebrows, and the extent of the scar indicated that the muscle had been implicated even to the conjunctiva; the light was painful, and the cornea had begun to lose its brightness. The operation was commenced by a moderately extensive incision in the middle of the remains of the upper lid, beginning about two or three lines distant from the inner canthus, and one line and a half above the palpebral margin. Fricke now allowed the eyelid to be put upon the stretch by an assistant, and finished the incision, which was carried in a curved direction at the required distance from the upper palpebral margin, where he commenced, through the skin to within about two lines above the outer canthus. The cellular membrane was next separated, and, together with the degenerated and condensed muscular fibre, dissected away, so as to leave bare the conjunctiva. The margins of the wound now gaped widely apart, and the upper lid receded. This was the period selected for taking the admeasurement of the requisite flap to be transplanted, and its outline was figured on the cheek. The next step was to raise the flap, taking care to leave a sufficiently wide neck of connexion at the point of twisting; and it was then fitted to the edges of the wound in the lid, so as to be adapted accurately to the separated margins." (*Zeis*, pp. 357-9.)

At the completion of this case the patient was extremely improved; the cornea having recovered its transparency, and the conjunctiva lost its swollen and injected state. The new eyelid fulfilled its office perfectly.

The method proposed by Dieffenbach, which is the most perfect operation, is detailed by him in *Casper's Wochenschrift* for 1835, (No. i. p. 8,) and, we are informed by Dr. Zeis, has been practised with success by Chelius, Eckström, Lisfranc, &c. We say it is the more perfect operation, because, in Fricke's method, it is essential that the original palpebral margin should be available in forming the new lid; whereas, in the Berlin professor's method, the restoration is complete. We shall paraphrase our author's account of this, as of the former operation; premising that, when the condition of the conjunctiva is tolerably sound, it is dissected up from the remnants of the former lid, so as to be employed as a lining for the fresh one. Of course, the preexistence of any malignant affection, such as carcinoma, forbids this reservation of what might communicate disease to the new structure: the advantage, however, of the modification, when practicable, is self-evident. The operation is commenced by two incisions, one extending from either commissure of the tarsus, which are so inclined towards each other as to meet at an acute angle—on the cheek when the lower lid is the object of restoration, and above the eyebrow when the upper lid is defective: this triangular flap, of which the third side is the remnant of the former lid, is then dissected up, and completely removed; care being taken to spare, as much as possible, the neighbouring nervous filaments. The space thus left is that intended to be occupied by the transplanted flap. The next step, whether for upper or lower lid, is to carry a horizontal incision from the external canthus over the zygoma, and in a straight line towards the external auditory meatus; and this incision must, in any case, exceed in

* Die Bildung neuer Augenlieder nach Zerstörungen. Hamburg, 1829. 8vo.

length the breadth of the defect in the eyelid. From the extreme outer point of this incision another is carried (when the lower lid is required) downwards upon the cheek, and for the upper lid upwards upon the temple. In either case, this incision must be nearly parallel to the outer line of the removed flap; its termination being on a level with, though slightly approximated to, the point of the same. Here, then, we have the means prepared for replacing the lid. The new flap is gently raised, and, after a careful cleansing of the space prepared for it, removed to its new position. The twisting of the broad pedicle, which is thus placed inferiorly, is very slight in this operation; for that which formed the superior margin of the flap becomes the edge of the new lower lid; the converse being the case in the upper operation. The same course, as regards sutures, is pursued in this as in the rhinoplastic operation. "So perfect," concludes Dr. Zeis, "has been the restoration in these cases, that Dieffenbach has of late years operated on numerous individuals for both defects, in whom the difference between the natural and artificial eyelid could not be detected." (pp. 360-3.)* A similar form of operation has been practised by the same surgeon, for complete ectropium with eversion of the outer commissure. He excises this latter portion, together with a triangular piece of the neighbouring integument of the temple, one side of it facing inwards, and one angle pointing towards the ear; a curved incision is then carried above the supra-orbitar arch, and another, of an inch and a half in extent, beneath the lower orbitar margin, and towards the nose. Both of these crescent-shaped flaps are then raised, and, after closing the temple-wound, are adapted as new lids to the remaining conjunctiva. (p. 364.) The ordinary operation resorted to by Dieffenbach for eversion of the lower lid will be rendered intelligible by the accompanying illustration. The triangular flap in the left-hand figure being raised, the sections *a*, *a* are extended freely on either side, to allow of the ready approximation of the two sides, *b*, *b*: these being then fixed by suture, the two cut margins, *a c* and *c a* are connected to the corresponding margins of the lower lid included between *c*, *c*. The whole lid is thus raised, and the parts are made to assume a natural and healthy character. (*Zeis*, p. 378.) Blandin does not appear to have been so fortunate in his blepharoplastic operations, even according to his own account; a fact which tempts Dr. Zeis to remark, that he had better have refrained

* The description of a similar operation, proposed and practised by Dr. Ammon, of Dresden, may be read by turning to Vol. IV., p. 483, of our Journal; where also will be found an illustrative woodcut of the same.

from narrating one of his cases altogether. He, however, relates an instance in which Jobert operated successfully for restoring a lower lid, after the removal of the original one in a cancerous state: the flap was derived from the malar region. (*Blandin*, p. 64.)

We would fain proceed further with a detailed analysis of the present chapter, which contains many interesting modifications of the above operations, for various forms of ectropium, entropium, lagophthalmos, &c., by Dieffenbach, Jäger, Von Ammon, and others; but our limits forbid us entering more at large upon the subject of complex operations, which require little short of transcription to render them intelligible: we must therefore refer our readers to the original, whilst we proceed to a brief notice of the ensuing chapters.

The *Cheiloplastic* operation* is the next in succession treated of by Dr. Zeis, and may be strictly defined as that branch of our subject which is directed to the restoration of the upper or lower lip, exclusive of the treatment required for remedying various modified abnormal conditions, such as congenital defects, contraction of the mouth, &c. This form of operation is by no means of so late a date as that last described; but was, as formerly remarked, of ancient origin. Dr. Zeis first notices a misapplication of this operation in cases where the congenital defect of harelip is such as to leave a wide space between the separated portions; and justly remarks, that the deficiency is never so great but that the yielding condition and lax texture of the cheeks is such as always to admit of the far simpler remedial means of approximating and uniting the opposed margins of the fissure. "The most common cause of destruction of the lips," observes Dr. Zeis, "is real cancer, which usually attacks the lower lip, or some modification of this disease extending from the nose or commissure of the mouth over the upper lip." Accidents, and other forms of disease, may likewise give rise to a loss of structure, requiring the art of the surgeon to procure a partial or total restoration of these organs. Nor must it be imagined that the defect is simply one affecting the appearance or even comfort of the patient: the loss of the under lip is of the most serious import, as allowing the escape of the saliva. Taliacotius, in his operation for the restoration of the lips, procured his material from the same source as that by which he supplied the loss of the nose, viz. the arm. But we shall pass over his and the Indian method, to notice the more recent modes of performing the operation. This branch of plastic surgery has received the attention of many eminent surgeons, amongst whom we may enumerate Delpech, Dupuytren, Lynn, (who, as Mr. Carpue informs us, imitated the Indian operation;) and, in the present day, of Velpeau, Roux, Dieffenbach, and many of our own countrymen. The operation practised by Chopart consisted of separating and drawing upwards the integument of the chin for replacing the lower lip; a method which, though simple, is obnoxious to the disadvantages resulting from after-contraction. Dupuytren's operation was an improvement on this, inasmuch as he liberated the integument of the cheeks by free incisions, for the purpose of facilitating the removal of the required flaps: and his success was proportionably great, as we learn from a case detailed in his "Leçons orales de Clinique chirurgicale,"

(tom. i. p. 25.) Roux has practised a further modification, by removing the prominent centre of the lower jaw, to allow of the more ready elevation of the integument covering it; and he conceives that an analogous operation in the upper maxilla might be desirable and practicable. After noticing these and other forms of operations for effecting the same result, Dr. Zeis at length ushers in the method adopted by Dieffenbach, with even more than the ordinary allowance of flattery, which, however merited, must, in such doses, be rather nauseating to the Berlin professor; "for," says our author, "had nothing more than what I have already related been done for the cheiloplastic operation, it would indeed stand low: but, as plants thrive in the hand of the gardener, so is it likewise with this and with all other plastic operations which Dieffenbach has undertaken, and which have received such important improvements from him." (p. 419.) The improvements alluded to are these: that, with his accustomed boldness, Dieffenbach divided not only the integument but the whole cheek in his lateral incisions; and that, secondly, by a form of operation analogous to that described in the last chapter, for restoring the eyelids, he reproduced lips, not of skin alone, but consisting of sections of the cheeks, and therefore endowed with all the requisite constituents of mucous membrane, muscle, &c. This latter operation we shall give according to Dr. Zeis's description.

"Having pared away the useless remains of the former under lip, or refreshed the cicatrized margin, a horizontal incision, about two inches long, is carried from either angle of the mouth outwards through the cheeks, so as to throw the mouth widely open. The length of these incisions must be regulated according to the width of the mouth; or, as a general rule, the combined incisions must somewhat exceed in length the breadth of the upper lip. From the outer point of each of these, another incision is next carried obliquely downwards and towards the median line; the section in this case likewise extending through the whole thickness of the cheek. Thus, by means of the first operation for paring the cicatrix, and by the succeeding horizontal and vertical incisions, a flap will be prepared on either side to replace the defective lip: this flap is of a quadrangular form, and maintains a connexion of more than an inch wide with the soft parts covering the rim of the lower jaw. It may be useful further to separate the mucous membrane at its attachment to the gums, to allow of the more ready traction of the flaps." (p. 420.)

This is rather a fearful operation, considering the necessary injury done to the trunk of the facial artery, the division of large nervous filaments, and the probable, if not certain, section of the parotid duct; yet has Prof. Dieffenbach practised it with perfect success, and without permanent ill consequences. Mr. Liston gives a preference to the simpler operation of raising a flap from under the chin: he recommends that the connecting peduncle should be left "thick and fleshy," to ensure a more active circulation through the transplanted skin. "After adhesion has been completed," he adds, "the (twisted) attachment is divided, and as much removed, in a wedgelike form, as will admit of the lower part of the flap being laid down smoothly, when it is retained in close apposition with the subjacent parts, either by suture or bandage." (p. 235.) This operation was performed by Mr. Liston ten years since.

In connexion with the last subject, the next two chapters are occupied with the *Stomatoplastic* and *Meloplastic* operations,* which signify relatively the restoration of the *mouth* and *cheeks*. As Dr. Zeis remarks, "the right of the former to admission amongst the plastic operations may fairly be questioned, as the mouth may be said to hold a *negative* relation to the lips." The two subjects are, however, to a certain extent related; and, although the object of the stomatoplastic operation is precisely the reverse of most other forms of plastic surgery, viz. the enlargement instead of the closure of an opening, it must be admitted that it is as essential to have a mouth as a nose or cheeks; therefore will we go a short distance with our author in his description of the means proposed for remedying the defect, which may at any rate be termed *positive*. The contraction of the orifice of the mouth, or the adhesion of its margins, is a great deformity, as well as being a source of extreme inconvenience, especially when the amount of diminution in circumference is such as to interfere with the introduction of solid food; and, when combined with a depressed nose and obliterated nostrils, as is frequently the case where the common cause is syphilis, the aspect is rendered hideous and revolting. To the inexperienced in such matters nothing would appear easier than to remedy the above condition by simple incisions; yet practical surgeons are but too well acquainted with the obstinate tendency to contraction so often evinced by skin and cellular membrane which have been the subject of tedious or ill-conditioned forms of ulceration. Of such class are burns, syphilitic or phagedenic ulcers, &c., each the occasional source of contracted mouth: and to these we may add the abuse of mercury, as no infrequent cause of the destruction of soft parts about the mouth and nose.

Dr. Zeis adopts Diffenbach's division of the different modifications of this deformity, which he classes under three heads: "1. Adhesion of the inner surface of the lips and cheeks to the jaws. 2. Contraction of the outlet of the mouth, reducing the aperture to the form of a round hole. 3. Such destruction of the lips and loss of structure in the whole circumference of the aperture, as to leave the teeth denuded and prevent the separation of the jaws." (p. 436.) This last-mentioned form is of far less frequent occurrence than the others. In simple contraction of the orifice of the mouth, mechanical dilatation by bougies and similar instruments has been employed; but of these Dr. Zeis does not approve; "for they tend to produce great discomfort by exciting irritation and excoriation on the margins of the aperture, and cannot be of any avail, owing to the indurated condition of the parts affected; and when relinquished, the opening contracts even more rapidly than before." (p. 438.) In these simpler forms, where division alone might not be successful, Dieffenbach recommends that, in addition, a portion of the neighbouring mucous membrane should be detached from its cellular connexions, and drawn over the cut surfaces: being fixed in this position by sutures or needles, adhesion cannot take place.

This operation is so ingenious, and has succeeded so well in the hands of others, besides its distinguished originator, that the several steps of it are worth detailing. We suppose a case then, in which the aperture of the mouth has been reduced to a mere rounded orifice by the contracted cicatrix of an ulcerated surface. The patient being seated, the fore-finger of the left hand is introduced within the oval aperture, and pressed outwards: the margin of the opening being thus rendered prominent, is pierced by one blade of a pair of narrow, straight scissors, which is carried horizontally backwards and outwards external to the mucous membrane, and as far as it is desirable to enlarge the mouth on one side of the mesial line: the blades are then closed, and all the superficial structures, such as skin, cellular membrane, muscles, &c., are divided at one sweep. A second incision parallel, but inferior to the first, is next accomplished in a similar manner; the former must be on a plane superior, and the latter inferior to the commissure of the lips, the distance between the two being about three lines. These incisions are then united at their external extremities by a small crescentic section, and the thus isolated flap is dissected out. The jaws being now widely separated, the stretched mucous membrane is divided horizontally and midway between the two incisions, care being taken to leave sufficient undivided for reflexion over the new commissure. The flaps are lastly everted above and below, and fixed by suture so as to form an investment to the newly-opposed surfaces. The same course is then pursued on the opposite side of the aperture. Dr. Mütter, of Philadelphia, to whom we have already alluded, has successfully performed this operation.

Where the mucous membrane is internally adherent to a considerable extent, slices are removed from the cheeks by the aid of scissors, and the adhesions being freely divided, the mucous membrane contracts, and the parts are then readapted, the required aperture being left. For the third form, a most sanguinary operation has been proposed and successfully practised by Professor Dieffenbach, (Erfahrungen, Bd. 3, p. 110;) this is also given at length by Dr. Zeis, but is too long for insertion here: it consisted in paring away the ancient cicatrices, and replacing the soft parts from the integument of the upper and lower jaws, the requisite aperture being left for the mouth.

*Defects* of the *cheeks* are perhaps amongst the most difficult to remedy where the loss of structure is extensive. Much, however, may be done by drawing upon the surrounding soft parts, and by actual transplantation. Roux has practised a very ingenious mode of aiding the ordinary removal of skin: it consists in procuring the required portion from a distance, and gradually conveying it by separate operations to the defective spot. A case of this nature is narrated by Blandin, (p. 96,) in which a girl had lost a portion of the left side of the upper lip, the corresponding ala of the nose, and part of the cheek; the flap was borrowed from the lower lip, and being first attached to the upper lip, was subsequently transferred to the cheek: the deformity was thus removed.

On introducing the subject of *fistula lacrymalis*, Dr. Zeis remarks, that " the great variety of methods recommended for the cure of this complaint demonstrates the doubtful result of the operation. . . . . . The ordinary treatment by incision will frequently prove abortive, in consequence of the opening failing to close." (p. 456.) Thus, then, is the case for the plastic surgeon made clear; and after the failure of other remedial agents, Dieffenbach has recommended the following operation, which we shall illustrate by a case. A lady, who had been operated on six years previously for fistula lacrymalis, applied to him for relief, after every attempt to close the orifice had been resisted. The circumference of the aperture was red and swollen, a condition in which the conjunctiva generally participated, as well as the cheek from the irritation of the tears: the nasal duct was likewise impervious. This canal was first bored, and a wire passed through into the nose. Six weeks were now allowed to intervene, at the end of which period this style was removed, and the operation proceeded with. "A crescentic portion of integument was first detached from the inner angle of the eye; a semicircular incision was next made on the opposite side of the opening, and an oval flap thus produced, three lines wide and four long, the upper and lower extremities of which retained their connexion to the skin of the nose: the flap was then drawn over the fistulous aperture, in such manner that its posterior margin came in contact with the opposed side of the opening, where it was fixed by means of fine needles." The case was ultimately successful.

In order to remedy the unsightly appearance (we mean no pun) presented by the sunken eyelids after extirpation of the globe, Dieffenbach has recommended an operation for filling the cavity, which consists in dividing the outer commissure of the lids, and robbing the neighbouring portion of the temple and zygoma of its integument: these flaps are then placed in the orbit and made fast by sutures to the circumference of the lids; which latter are then closed over by strips of adhesive plaster, and the margins of the temporal wound are approximated. Of course this operation is not intended to supersede the employment of an artificial globe; nor can we consider it very applicable in any case.

The operation for the cure of fissured palate is omitted for the same reason that led our author to avoid mentioning that for harelip: we, however, have a short chapter upon the *restoration* of the *soft palate*, which is named the *Staphyloplastic* operation.* The case, related by Dr. Zeis, of a girl operated on by Dieffenbach did not terminate satisfactorily. The operation must of course be modified according to the amount of loss of structure; and can, in any case, even when successful, be but an imperfect substitute for the original palate.

The *Otoplastic* operation† occupies chapter ix. Taliacotius, as we formerly stated, practised the restoration of the ears, employing for that purpose the neighbouring skin of the mastoid region. We have but few recent examples of this form of plastic surgery. Dieffenbach recommends a similar operation to that adopted by the Italian Professor; and states that he has, in the same way, remedied partial loss of the ears. (p. 467.)

The *Bronchoplastic* operation‡ has, for its object, the closing of fistula in the trachea. The most successful, and therefore the most desirable, method is that recommended by Professor Velpeau, and consists, "1, in the formation of a flap, the figure of which is a rectangle drawn out, with its base separated some lines from the solution of continuity; 2, in the rolling of this flap on its *cutaneous* surface into a sort of plug which is to be introduced into the aperture to be closed." Velpeau remarks, that this form of operation is applicable to the cure of various kinds of fistula, apertures in the intestines, the radical cure of hernia,

* From Σταφυλὴ, a plummet, a grape: *met.* the uvula.

‡ From Βρογχος, the throat, the trachea.

&c. (*Blandin*, p. 154.) A case of laryngeal fistula is related by Blandin, which had resisted the ordinary form of plastic operation, but yielded to the above mode of treatment; the voice, deglutition, and respiration, which had been materially impaired or interfered with, being thus restored. (p. 160.) It is almost unnecessary to remark, that the edges of the fistulous orifice require paring.

In the Gazette Médicale, (1834, No. 42,) there is a paper by M. Martinet de la Creuse, in which he demonstrates the valuable properties of plastic surgery in preventing the return of cancer and other malignant diseases after removal of the parts affected. The cases he cites are extracted at length by both Zeis (p. 471) and Blandin (p. 229.) The nature of his operation was simply to supply a sufficiency of foreign integument to close the aperture left after excision of the diseased structure. Martinet permitted an interval to elapse, to allow of suppuration and granulation being established; but Blandin thinks it better to perform the transplantation at once. Both our German and French authors speak in a cautious manner regarding the general application of M. Martinet's deduction from the very limited data he possesses, viz. four cases.

In chapters xii. and xiii. of Dr. Zeis's work, we are taught the method of *restoring* the *prepuce* and *scrotum*. The former of these, termed the *Posthioplastic* operation,* dates its origin as far back as the period when the Jews, in the Roman empire, used to furnish themselves with fresh prepuces, in order to avoid the persecution attached to their race. The simplest form of operation is applicable to cases of naturally contracted or shortened prepuce; it consists in drawing forwards the foreskin until it covers the glans penis, and then making an annular incision through the integument posterior to it. Care should be taken not to injure the urethra, and a ligature must be placed around the prepuce so as to retain it in its new position, allowing only room for the urine to escape: the gaping wound must heal by granulation. (p. 480.) Where the prepuce is totally deficient, an annular incision must be made near the base of the glans, and the integument dissected up to the requisite extent to allow of the new foreskin being drawn forwards; in which position it must be retained after the inflammation has a little subsided, by means of a bandage applied around the root of the penis. The same operation is applicable after the removal, by circumcision, of an indurated and morbidly elongated prepuce.

Respecting the *Oscheoplastic* operation† we shall say but little. It is usually required either after spreading ulceration has exposed the testicles, or, as is more common, in cases where disease, such as elephantiasis, requires the removal of all the original scrotum. For this latter form of defect a complex operation was performed by Delpech, and more recently by Clot-Bey and Velpeau. (Zeis, p. 487, Blandin, p. 75.) Of simple exposure of the testicles from sloughing, we have witnessed more than one case in which nature has reproduced the whole scrotal tegument without the aid of plastic surgery. Labat states, that he has performed this operation by borrowing the integument from the thigh or from the belly. (Rhinoplastic, p. 336.)

† From ʺΟσχη or οσχέα, the scrotum.

The treatment of *prolapsed uterus* is included in the wide grasp of our extensive subject. In such cases as admit of a ready return of the womb, but resist mechanical means of retention, Dr. Zeis recommends the partial union of the nymphæ, as practised by Fricke, presupposing that there is no disorganization of the uterus, nor extensive destruction of the soft parts externally. (p. 501.) A similar result has been aimed at by the removal of strips from the vaginal mucous membrane, but with variable success. We have witnessed an analogous operation for prolapse of the bladder into the vagina, the issue of which was not very encouraging.

The *Urethro-plastic* operation (which occupies chap. xv.) is an important one, and therefore extends through several pages of Dr. Zeis's work. We shall notice some of the more recent operations for the cure of urinary fistula, which owe their chief improvements to Dieffenbach. The size of fistulous apertures, communicating with the urethra, differs very materially, varying from a caliber not more than sufficient to admit a bristle, to the diameter of a good-sized catheter; the flow of urine through them of course bears a proportion to their size: all are troublesome, but those situated near to the scrotum are the greatest source of annoyance to the patient. Dieffenbach remarks, that he never saw a small fistula communicate directly with the surface, as is the case with those possessing large mouths; the former usually pierce the canal obliquely, from behind forwards and outwards. The application of caustic washes, and the use of simple suture after paring the edges, are amongst the curative means which have been employed with occasional success: but, independently of their questionable issue, these methods are open to the objection of leaving the fistulous orifice more gaping than previously, and the hazard of producing considerable inflammation, and even infiltration of urine, as Dieffenbach has proved experimentally. (*Zeis*, p. 509.) Nor has this surgeon obtained better results from the employment of the twisted suture over a needle. Now, as the tension appeared to be in this, as in many other instances, the cause of failure, Dieffenbach has proposed and successfully practised the following operation. " An elongated, oval flap of integument, *a*, having the fistula in its centre, is removed, and the margins of the wound are then armed with a sufficient supply of sutures to close it; a simple lateral incision, *b, b*, is next made to the same extent, and at a short distance removed from either margin of the former, and the flaps raised from their central connexion: these are now approximated by tying the sutures, and a catheter is kept in the bladder ....... Nocturnal erections of the penis might thus occur without disturbance of the sutures, and although the urine would flow for a time by the side of the catheter, and make its escape by the lateral apertures, still the cure was usually completed in about a fortnight." (*Zeis*, p. 511.)

Where simple sutures have been found objectionable, small leather splints have been interposed on the inner margin of either flap, to act as a protection and to preserve the parts in better apposition. Some of our surgeons have successfully practised simple transplantation of integument for closing fistulæ; and, according to Blandin (p. 180), M. Alliot recommends this form of operation in preference to that of Dieffenbach, as he is of opinion that the introduction of the urine between the interstices of the sutures is the most common source of ill success. Blandin himself gives the preference to Velpeau's plan of plugging the orifice by a roll of skin, as already described for the cure of tracheal fistulæ. Delpech, in his "Clinical Surgery," relates a case in which he performed the simple transplanting operation, deriving his flap from the inguinal region; but gangrene succeeded, and the cure was only partial. The cause of the fistula was, in this instance, curious. The patient, a young lad, was in the habit of wetting his bed at night, for which, being menaced with severe treatment by his parents, he devised the plan of placing a ligature about his penis: but not taking the precaution of including the prepuce alone or of graduating the pressure, the result was, between this and the consequent swelling, that the string cut its way into the corpora cavernosa and urethra. We have several other methods of closing urethral fistulæ cited by Dr. Zeis,—such as, twisting round the whole skin of the penis upon its axis (515;) the annular removal of the fore-skin backwards, for closing a fistula immediately behind the prepuce (517;) a similar transplantation of the skin of the penis forwards, where the prepuce is deficient to close a fistula immediately beneath the glans (518;) ; and, lastly, the slip or running suture by which the mouth of the fistula is closed like a bag, as we had occasion to describe a few pages back: this last operation possesses the prominent advantage of leaving the patient, in case of its failure, not worse off than he was before the experiment. For further details we must refer our readers to the original works.

The term *Cystoplastic* operation,* has been indiscriminately, and therefore incorrectly, applied to all forms of surgical assistance for the cure of fistulous openings communicating with the bladder: it should correctly be limited to the cure by translation of skin from a neighbouring part. Of this true cystoplastic process Dr. Zeis notices the method adopted by Delpech, for the cure of congenital inversion of the bladder. To this end an elliptical flap of skin was raised from the hypogastric region, and fixed to the pared margin of the gaping aperture, care being taken not to include the mucous membrane of the

* From Κύστη, or κύστις, a bladder; the urinary bladder.

bladder in the sutures: a catheter was retained in the urethra till the cure was completed.

Of all the miserable conditions we have had occasion to allude to in the foregoing pages, perhaps no one exceeds, and few equal, the wretched state to which a patient is reduced by *vesico-vaginal fistula.* Of this a vivid picture is drawn by Dieffenbach, (*Med. Zeitung*, No. 25, 1836,) the faithfulness of which we must reluctantly admit. " Such unhappy beings," he observes, " are forced to exclude themselves from society; the very atmosphere surrounding them is polluted by their presence, and even their children shun them: thus rendered miserable, both morally and physically, they yield themselves a prey to apathy; or a pious resignation alone saves them from self-destruction." (*Zeis*, p. 526-7.) All mechanical means for palliating this wretchedness have proved of little or no avail: and the employment of the simple suture has rarely been attended with happy results; for the presence of the urine mars all efforts at adhesion or granulation. The ordinary sources of this form of fistula are ascribed by Dr. Zeis to " difficult labours, and the rough use of mechanical aid in the same; injuries done in passing the catheter, or by bad pessaries; stone in the bladder, lithotomy, abscess, &c., are further causes of these fistulæ, which are occasionally complicated with prolapse or carcinoma uteri, fistula or stricture of the rectum, &c." (p. 525.) Dieffenbach's running suture, to which we have more than once alluded, has been frequently and successfully employed by its inventor, and is that form of operation which is preferred by him, although he admits that its employment must not be indiscriminate, and that by it the desired object is not always obtained. Two unsuccessful cases are related by Zeis, one of the patients dying from inflammation of the bladder. M. Jobert has had some success in closing a vesico-vaginal fistula by the true plastic method: " this operation consisted," says Blandin, " in paring the edges of the fistulous orifice, and adapting over it an oval flap derived from the internal surface of the larger labia." The case related at page 83 was partially successful. In another instance, much inconvenience was experienced from the aftergrowth of hair on the transplanted flap.*

The plastic treatment of *artificial anus* is passed over in one page. A case is quoted from Gräfe and Walther's Journal, (Bd. ii. p. 655,) in which a perfect cure was affected by simple transplantation of integument over such an opening which had been made in a hernial tumour, by a surgeon who mistook the swelling for an abscess. Dieffenbach is of opinion that his running suture is applicable to these cases.

Lastly, we have chap. xviii. devoted to the means of radically curing hernia by a plastic operation; but we treated so fully of this in our Twelfth Number (p. 341), that we may pass it entirely without notice here.

* We had occasion lately to witness a case which would have puzzled even the ingenuity of Dieffenbach to cure. It consisted in destruction, by ulceration, of the whole urethra, the aperture thus left allowing of the free ingress of the little finger: the incontinence of urine was therefore established. Yet the neck of the bladder and the annular layer of muscular fibre surrounding it were, apparently, undisturbed; a circumstance which, taken in conjunction with many other facts, renders it highly probable that the so-called sphincter has little or no share in retaining the urine either in man or woman.

Thus have we arrived at the conclusion of our subject, although not quite at the end of Dr. Zeis's volume: he has still another chapter devoted to the *division of muscles and tendons* for the purpose of remedying deformities resulting from their permanent contraction. The analysis of this chapter we intentionally omit, because it is not essentially a part of the subject we have been discussing, and because we promise ourselves an opportunity of recurring to it separately and more at length than our present limits would admit.

We have but little to say respecting the relative merits of the two works which have formed the principal basis of the foregoing article; indeed they scarcely bear comparison. That of Dr. Zeis is unquestionably the more perfect and better arranged, although vastly more bulky than is essential for even a full discussion of the various branches of plastic surgery; but this diffuseness is too generally cultivated to be considered a fault in Germany. The cases are given too much in detail by both authors; and this is the more reprehensible in Blandin, who professes to have compressed his matter into as small a compass as possible. We certainly cannot tax Dr. Zeis with having treated his subject superficially, a charge which, in his preface, he makes against his French contemporary, in rather an ungenerous manner: indeed, both here and in many parts of his work, as we have more than once had occasion to observe, the contemptuous tone and very national ill feeling evinced by Dr. Zeis towards M. Blandin and his countrymen is, to say the least, uncourteous and unbecoming a man of science. The above characteristic is not rendered less prominent by the fulsome flattery with which his own countrymen, especially Dieffenbach, are loaded, a *quo* which was not, however, without its *quid;* for we find the Berlin Professor penning, in return, a recommendatory preface of a page and a half, and allowing (are we right in so supposing?) under this excuse his name and its appendages to appear in the title-page. We should think such a book-selling job would scarcely be permitted to pass unnoticed by the German press. We have only to add, that Dr. Zeis's work is embellished by numerous and very useful illustrative woodcuts, and two coloured engravings representing the condition of a new-formed nose in its various stages, passing into a state of gangrene. At the conclusion of his work, M. Blandin gives a recapitulation of the results of eighty-four cases, including the various forms of plastic surgery: but these data are too limited, and the deductions therefore too vague and unsatisfactory, to render such summary either interesting or useful. We have already, on more than one occasion, expressed our opinion of Mr. Liston's treatise, and need not, therefore, repeat it here. The work of Professor Dieffenbach has also been some time in the hands of the profession, and abounds in that originality of conception and boldness which characterize the practice of its author.

## Figures and Tables

**Figure f1:**
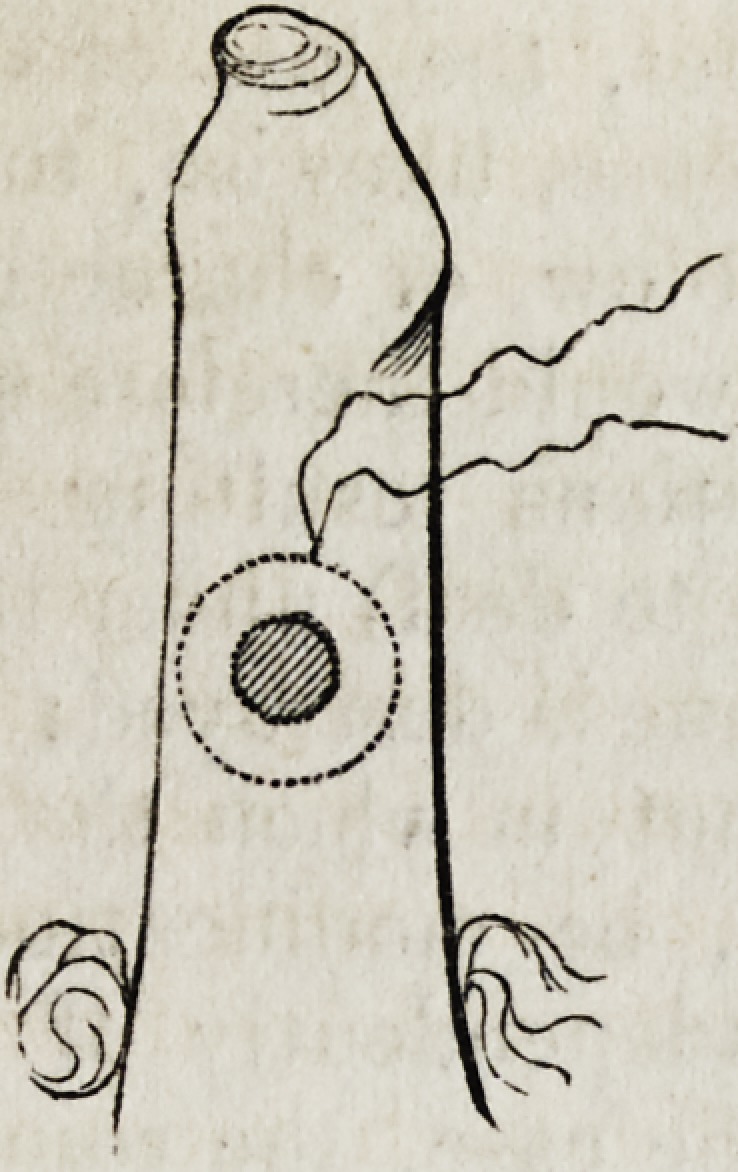


**Figure f2:**
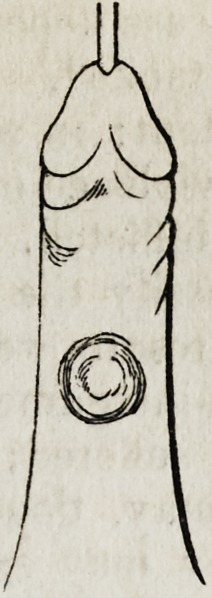


**Figure f3:**
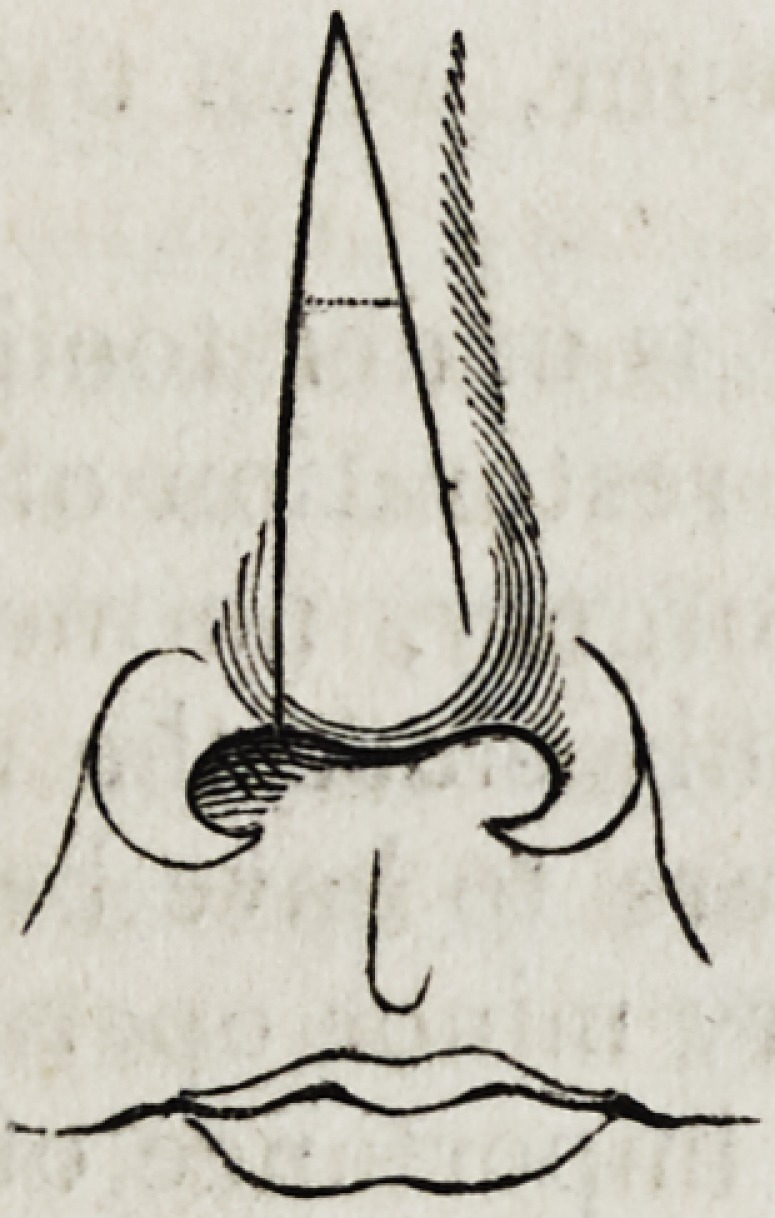


**Figure f4:**
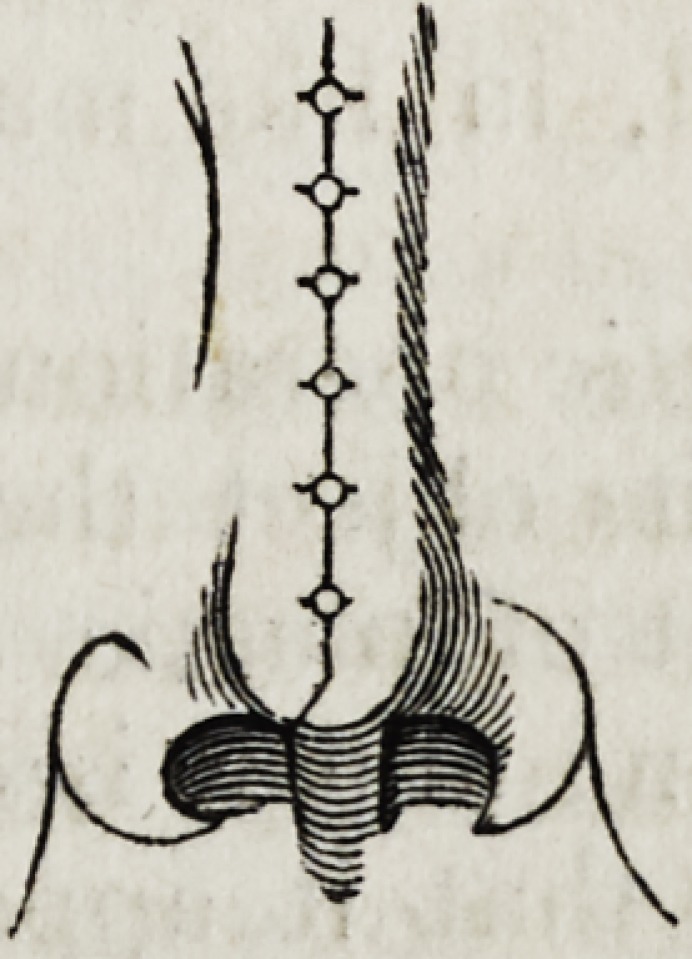


**Figure f5:**
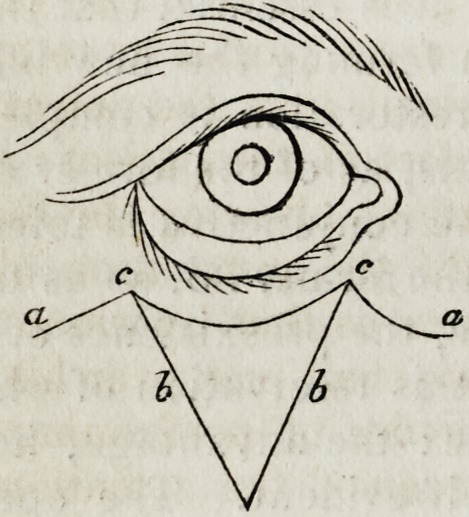


**Figure f6:**
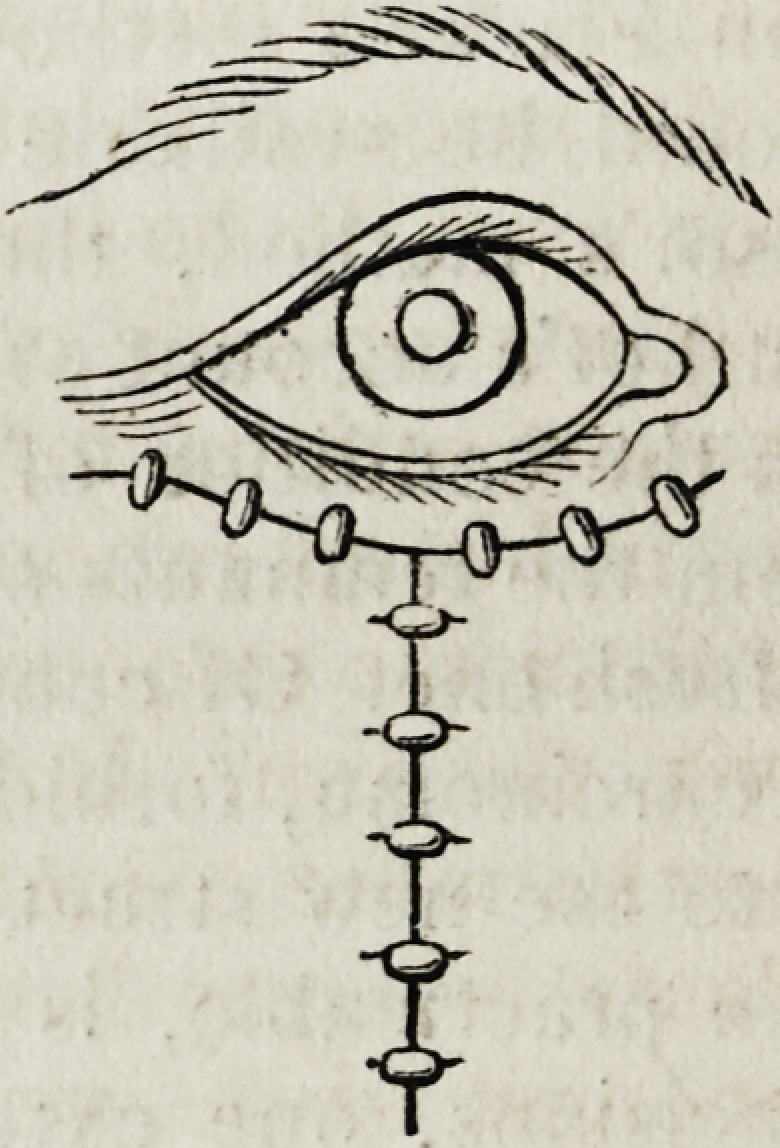


**Figure f7:**
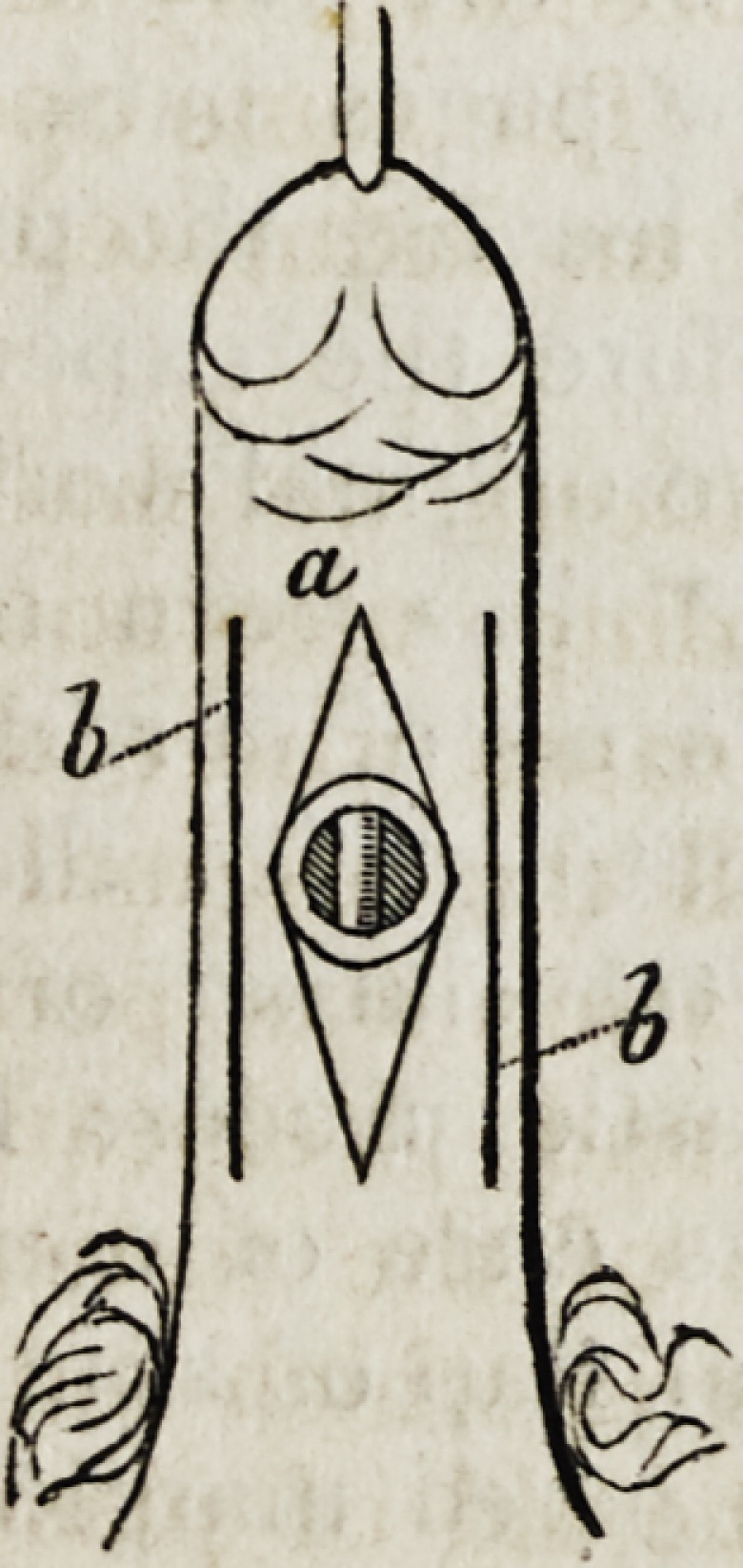


**Figure f8:**